# Exfoliation Energy as a Descriptor of MXenes Synthesizability and Surface Chemical Activity

**DOI:** 10.3390/nano11010127

**Published:** 2021-01-07

**Authors:** Daniel Dolz, Ángel Morales-García, Francesc Viñes, Francesc Illas

**Affiliations:** Departament de Ciència de Materials i Química Física & Institut de Química Teòrica i Computacional (IQTCUB), Universitat de Barcelona, c/Martí i Franquès 1-11, 08028 Barcelona, Spain; danieldolz1997@hotmail.com (D.D.); angel.morales@ub.edu (Á.M.-G.); francesc.illas@ub.edu (F.I.)

**Keywords:** MAX phases, MXenes, density functional calculations, exfoliation energies, synthesizability, chemical descriptors, CO_2_ capture

## Abstract

MXenes are two-dimensional nanomaterials isolated from MAX phases by selective extraction of the A component—a *p*-block element. The MAX exfoliation energy, *E_exf_*, is considered a chemical descriptor of the MXene synthesizability. Here, we show, by density functional theory (DFT) estimations of *E_exf_* values for 486 different MAX phases, that *E_exf_* decreases (i) when MAX is a nitride, (ii) when going along a metal M component *d* series, (iii) when going down a *p*-block A element group, and (iv) when having thicker MXenes. Furthermore, *E_exf_* is found to bias, even to govern, the surface chemical activity, evaluated here on the CO_2_ adsorption strength, so that more unstable MXenes, displaying larger *E_exf_* values, display a stronger attachment of species upon.

## 1. Introduction

Since their first isolation in 2011 [1], MXenes, a new family of two-dimensional (2D) layered materials, have constituted an incredibly growing hub of research on different fields of technology; from materials for Lithium batteries [2,3] to supercapacitors [4,5], electromagnetic interference shielding materials [6,7,8], and uses as heterogeneous catalysts [9,10,11] or biosensors [12,13], to name a few. Indeed, MXenes are part of the post-graphene 2D materials, which still offer new examples and applications, e.g., for CO_2_ utilization [14], as catalysts [15], or as new thermoelectric materials [16], to cite some. A particularly appealing field is the use of MXenes in environment-related applications, e.g., pristine MXenes, have been first theoretically [17] and later experimentally [18] appointed to be well-suited materials for carbon dioxide (CO_2_) greenhouse gas abatement, with CO_2_ uptakes an order of magnitude larger than other tested porous materials. This paramount performance is due to the MXenes inherently enhanced chemical activity, arising from their intrinsic metastability. Interestingly, density functional calculations have shown that MXenes can be regarded as the 2D versions of rocksalt transition metal carbides (TMCs) and transition metal nitrides (TMNs) [19], where the MXene (0001) basal plane is morphologically and chemically resembling the TMCs and TMNs (111) surfaces, planes normally not exposed given their high surface energies [20,21].

Many of the above applications imply the interaction of a given species with the MXene surface, thus the surface chemical activity being, a priori, a key feature. In this line, surface energies, also known as surface tensions, have been appointed as descriptors of the material’s chemical activity [22]. Such descriptors are aimed to provide quantitative predictions of physicochemical properties determining the feasibility of a given chemical process, e.g., the adsorption energies of atoms or molecules attached to the MXenes basal surfaces. The utilization of energy-related descriptors contrasts to the more extended use of electronic-structure-based descriptors [23], such as the *d*-band center in transition metals [24], better than other possible descriptors such as the width corrected *d*-band center or the highest Hilbert transform peak [23]. In O-terminated MXenes, the *p*-band center has been already used as a descriptor of the electrocatalytic hydrogen evolution reaction [25].

**Figure 1:**
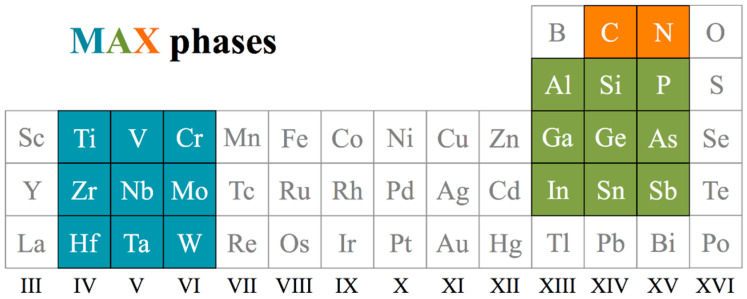
Section of the *d*- and *p*-block elements of the periodic table, where elements considered for the presently studied MAX phases and MXene are highlighted in color.

MXenes have M*_n+1_*X*_n_* composition, M normally being an early transition metal and X = C or N; see Figure 1. Notice that *n* defines the material’s thickness, normally *n* = 1–3, although MXenes with *n* = 4 thickness have been recently reported [26]. MXenes are obtained by selective extraction, i.e., using hydrofluoric acid (HF), from the so-called MAX phases, where A is normally a *p*-block element, see Figure 1. The intrinsic MXenes 2D nature does not allow acquiring estimates of surface energies since TMCs and TMNs are not appropriate bulk references. A suitable estimation of the MXenes stability can be gained through exfoliation energies measures, here defined as in the earlier study by Khazaei et al. [27]; that is, using bulk A elements as a reference in energy (see below).

Indeed, it is known that harsher etching conditions, in terms of HF concentration, temperature, and exposure, are needed when the bond of A element to the MXene layers is stronger [28,29]. Thus, *E_exf_*, capturing such interactions, are proposed to determine whether a given MXene is more or less easily achievable, i.e., to seize whether smaller *E_exf_*, and so weaker interactions among A and M elements, imply a larger MXene stability, and, hence, ultimately provide easier extraction conditions. Furthermore, a larger MXene stability is likely to imply a smaller surface chemical activity, as the surface would not require compensating the surface inherent instability by creating strong bonds with adsorbates. In the present work, by using density functional theory (DFT) calculations on a series of MXene and MAX models, we actually show that such exfoliation energies are well-suited descriptors of both the MXenes synthesizability and of their surface chemical activity, exemplified here on the CO_2_ adsorption, and thus related to the CO_2_ abatement.

## 2. Computational Details 

The exfoliation energies, *E_exf_*, have been acquired following previous definitions [27], so that

**Figure 2:**
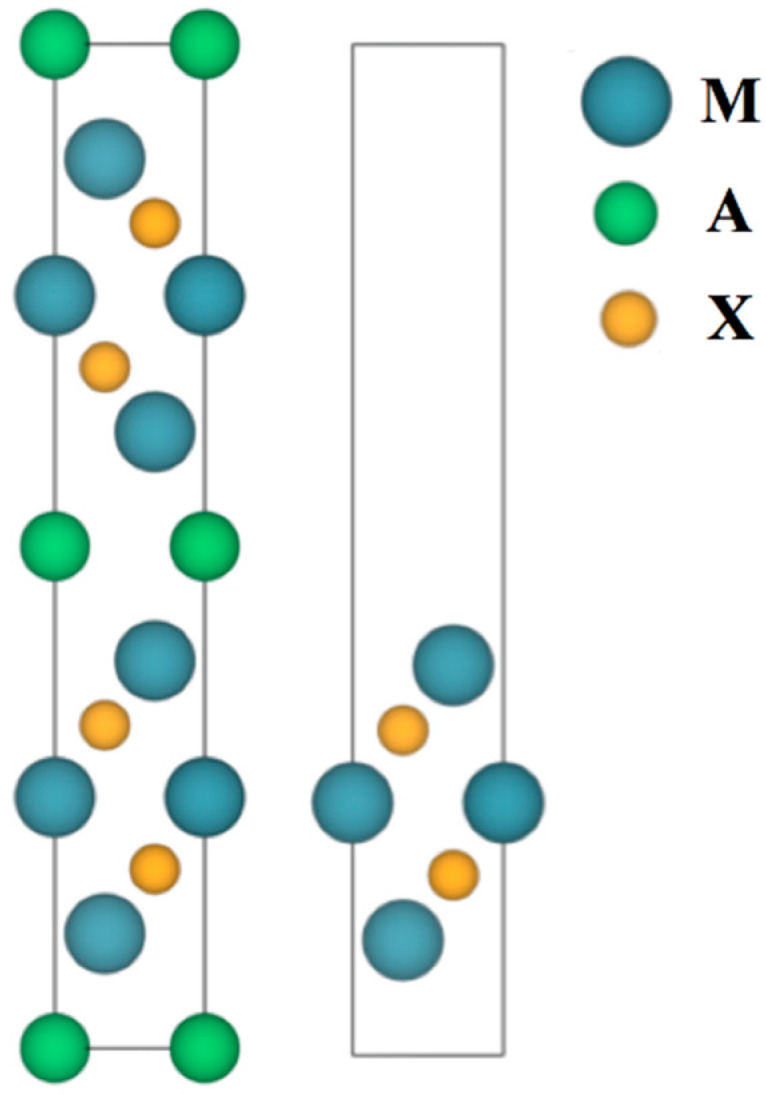
Side views of a MAX crystal cell (*left* image) and its derived MXene slab cell (*right* image). Notice the alternate disposition of the two MXene units within the MAX crystal structure. Blue, green, and orange spheres denote M, A, and X elements, respectively.

*E_exf_* = (2 · *E_MXene_* + 2 · *E_A_* − *E_MAX_*)/4 · *S_A_* (1)

where *E_MXene_* is the isolated MXene energy, *E_MAX_* is the MAX unit cell energy as shown in Figure 2, *E_A_* is the energy of an A atom in its bulk phase, and *S_A_* is the cross-section area of each created MXene unit. As observed in Figure 2 below, the MAX phase contains two MXene slabs and, therefore, four cross-section areas are created after the exfoliation process. Here, a systematic study has been performed considering all regular MXenes with *n* = 1–3 widths, combining M metals from groups IV–VI, and X being C or N; see Figure 1. Notice that a given MXene can be obtained from different MAX phases, and so, nine possibilities for the *p-*block elements are considered, from groups XIII–XV, see Figure 1.

The energies in Equation (1) have been acquired by first-principles periodic DFT calculations, carried out using the Perdew–Burke–Ernzerhof (PBE) [30] exchange-correlation functional as implemented in the Vienna Ab Initio Simulation Package (VASP) [31], known to deliver reliable estimates of the energetics in transition metal carbide bulk systems [32], as well as on the MXenes 2D counterparts [17,19]. The effect of core electrons on the valence electron density has been described using the Projector Augmented Wave (PAW) method [33], and the valence electron density expanded in a plane-wave basis set with a cutoff energy of 415 eV. Numerical integrations were carried out in the reciprocal space using optimal Г-centered Monkhorst–Pack *k*-points grids of 9 × 9 × 9 and 5 × 5 × 1 dimensions for MAX bulks and MXene surfaces, respectively [34]. The atomic structures of the A bulk elements have been fully optimized in their most stable crystal polymorph at ambient conditions, being face-centered cubic (fcc) for bulk Al (Fm$\overset{-}{3}$m space group); orthorhombic (Cmce) for As and Ga; cubic (Fd$\overset{-}{3}$m) for Ge, Si, and Sn; trigonal (R$\overset{-}{3}$m) for In and Sb; and triclinic (P$\overset{-}{1}$) for P [35], all optimized in the same fashion as for MAX bulk phases. In all cases, the electronic density convergence criterion was set to 1·10^−6^ eV, while the geometry optimizations were considered converged when forces acting on the relaxed atoms were all below 0.01 eV·Å^−1^. In the case of the isolated MXene slabs, we used previously employed *p*(1 × 1) cells [10,13,17,19], with a vacuum region of at least 10 Å to avoid interaction among periodically repeated slabs. This computational setup has been found to deliver energetic results within the chemical accuracy of ~0.04 eV.

In all cases, bulk cell dimensions and atomic positions were fully allowed to relax. In the case of MXenes, their 2D cell dimensions were initially adjusted to the parent MAX phase, yet fully relaxed—that is, accounting for variations in *a* and *b* cell vectors in the absence of the A element. However, the results revealed very small variations that could, de facto, be neglected. MAX and A bulk phases, as well as most of MXene layers, were calculated non-spin polarized, except, as earlier found for Cr-based MXenes [27]. Notice that small spin polarizations were earlier found for Ti- and Zr-based MXenes [36], but their treatment effect on the computed energies has been found to be negligible. Furthermore, the MXenes have been computed in the parent MAX phase ABC stacking, even if some MXenes are found to be energetically more stable adopting an AB stacking [36]. This is done as the initial exfoliation energy, and by that, other possible posterior layer reorganization processes have been placed out of the current analysis. 

## 3. Results

The presently calculated exfoliation energies are listed in Table 1, Table 2 and Table 3, accounting for a total of 486 situations. From these, 16 cases that were previously addressed by Khazaei et al. [27] reveal an almost perfect consistency of results, with an almost negligible average deviation below 0.04 J·m^−2^. The values for the thinner M_2_X MXenes, shown in Table 1, reveal *E_exf_* values ranging from 0.77 (W_2_SbN) to 3.72 J·m^−2^ (Ti_2_PN). Even in spite of these limits, by flicking through the listed values in Table 1, one already detects that N-based MAX phases display smaller exfoliation energies and that certain A elements, such as P, tend to deliver higher *E_exf_* values, and others, such as Sb, smaller *E_exf_* values.

The above analysis is generally mirrored for thicker MXenes, as obtained from M_3_AX_2_ and M_4_AX_3_ MAX phases, whose *E_exf_* values are encompassed in Table 2 and Table 3, respectively. The computed estimates again range from 0.26 (W_3_SbN_2_) to 3.65 J·m^−2^ (Ti_3_PN_2_), being somewhat smaller than M_2_AX derived ones. As far as M_4_AX_3_ cases are concerned, deviations are observed, as values range from 0.5 J·m^−2^ (W_4_SnN_3_) to 3.69 J·m^−2^ (Hf_4_PC_3_), thus even increasing from the intermediate MAX cases. Note that the negative value of −0.18 J·m^−2^ W_4_SbN_3_ indicates that such a MAX phase is thermodynamically unstable and would naturally decompose into Sb and W_4_N_3_.

**Figure 3:**
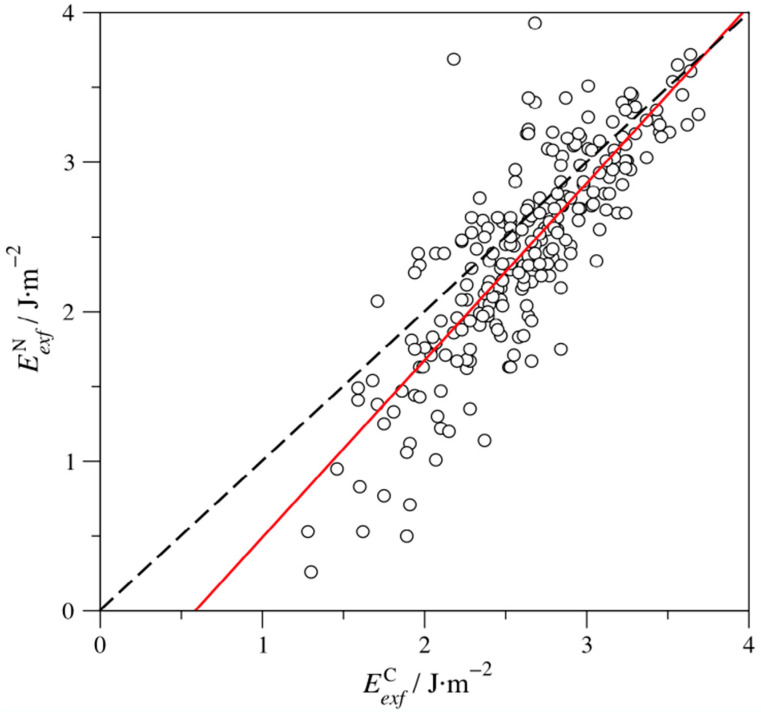
The linear trend of N-based MAX phases exfoliation energies $(E_{exf}^{N}$) with respect to the C-based energies ($E_{exf}^{C}$). The dashed line represents an ideal situation where all $E_{exf}^{N}=\;E_{exf}^{C}$. The red line represents the linear adjustment of the values, with a regression coefficient, *R*, of 0.83, and following the formula $E_{exf}^{N}=\;1.18\cdot E_{exf}^{C}-0.63$.

Excluding the aforementioned W_4_SbN_3_ case, one can argue whether certain MAX phases display larger/smaller *E_exf_* values. As far as the X component is concerned, Figure 3 shows that *E_exf_* for N-based MAX phases ($E_{exf}^{N}$) are generally smaller than C-based values ($E_{exf}^{C}$). Note that this trend is not systematic, as there are cases where the opposite relation applies. Besides, the smaller exfoliation energies for N-based MAX phases are more acute whenever the *E_exf_* values are small, and, actually, the divergence vanishes for large *E_exf_* values. All in all, the lower *E_exf_* values for N-based MAX phases is apparent from the hundreds of studied cases, but this should be treated more as an indication than a rule. 

**Figure 4:**
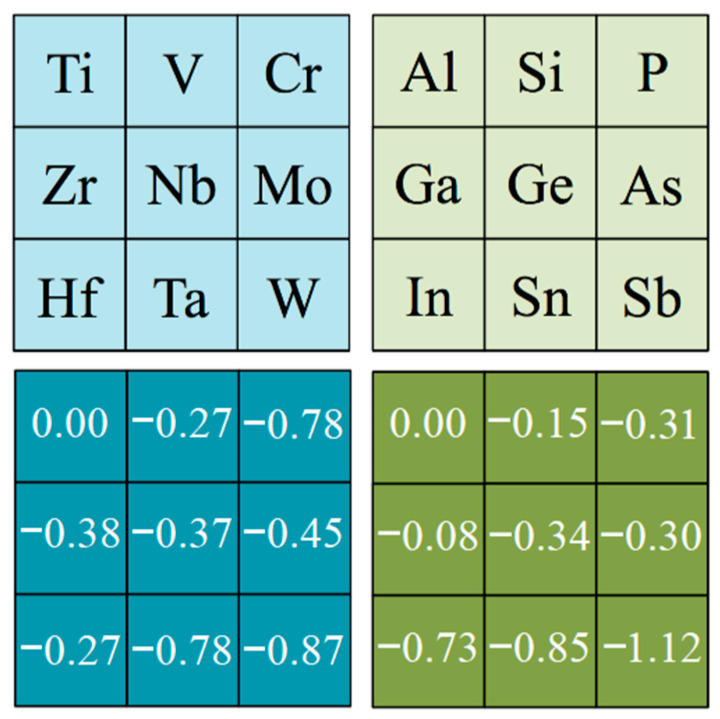
Mean deviations (MD) values for the computed *E_exf_* energies, taking either Ti-based MAX phases as reference in the M effect analysis (*left* panels, in blue) or Al-based MAX phases as reference in the A effect analysis (*right* panels, in green). All values are given in J·m^−2^.

Similarly, the effect of MAX phases being composed by a particular M metal or an A *p*-block element was investigated. Here, the Ti- and Al-based MAX phases were used as references for evaluating how *E_exf_* values changes by changing the M or A element, respectively—mostly because Ti_3_C_2_ MXene was the first-ever isolated, achieved by starting from the Ti_3_AlC_2_ MAX phase [1] and normally constituting a reference case for posterior studies. This accounted for, the Mean Deviations (MD) values were acquired for the exfoliation energies, grouped by families of MAX phases. Figure 4 shows the signed deviation according to these families, being nominally zero for Ti-based MAX phases when analyzing the M effect, and zero for Al-based MAX phases when analyzing the A *p*-block element effect. Thus, analyzing the M effect, one can detect some trend as the exfoliation energies clearly decrease when going along a *d* series, particularly for 3*d* and 5*d* metals, and somehow the *E_exf_* values decrease as well when going down a group, particularly true for group VI metals. At the same time, the analysis reveals that Al-based MAX phases tend to display higher exfoliation energies, and those W-based tend to display the lowest. This is probably due to the particular A-M bond in MAX phases, although a detailed electronic structure analysis is required to confirm this hypothesis. Such analysis is, however, out of the scope of the present study and will be placed in an oncoming study.

When it comes to the A *p*-block element effect, trends are less evident; one observes that the aforementioned *d* series decrease of *E_exf_* values holds for the fifth series only, composed of In, Sn, and Sb. On the fourth and third series, no particular trends were captured, i.e., with upside-downs, or about constant decreases. However, a clear, consistent trend was captured when going down the groups, as the *E_exf_* values decreased with no exceptions. Further than that, note that P-based MAX phases show, in average terms, higher exfoliation energies than Al-based MAX ones, and so, the extreme situations here would belong to P-based MAX phases featuring the highest *E_exf_* values, while Sb-based ones display the smallest values in mean terms. As far as thickness is concerned, taking *n* = 1 as reference, the MD for *n* = 2 and 3 were −0.13 and −0.22 J·m^−2^, thus being a mild factor, which goes in favor of little variations of the surface chemical activity, as observed for CO_2_ adsorption [19] and discussed below. In any case, altogether, the above analysis points out that C, P, and Ti with the smallest thickness are key ingredients for larger exfoliation energies and W, Sb, and N with the largest thickness are key ingredients for the lowest exfoliation energies.

**Figure 5:**
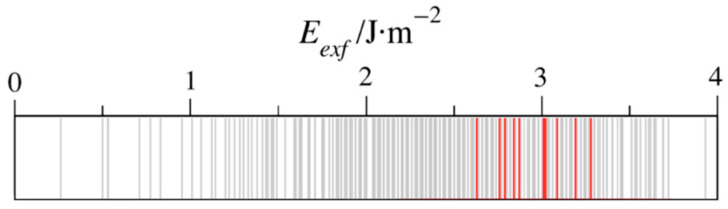
Display of all the computed *E_exf_* values in grey, while those MAX phases from which MXenes have been isolated [37] are highlighted in red.

An open question is whether the exfoliation energy is a descriptor of the possible MXene synthesizability. As mentioned above, previous studies indicated that the interaction among M and A elements, here seized by the *E_exf_* values, define how harsh the conditions of extraction must be [28,29]. Figure 5 orders the calculated exfoliation energies, with the experimentally successful cases highlighted in red, as recently collected [37]. These are Ti_2_AlC, V_2_AlC, Nb_2_AlC, Ti_2_AlN, Ti_3_AlC_2_, Ti_3_SiC_2_, Ti_4_AlN_3_, V_4_AlC_3_, Nb_4_AlC_3_, and Ta_4_AlC_3_. The predominant components are Ti, Al, and C with *n* = 2, which, surprisingly, imply components with larger exfoliation energies. Therefore, this highlights the possibility of isolating MXenes from other MAX phases under milder conditions [20]. One has to keep in mind that other factors affect the MXene synthesis, such as the MAX phase intrinsic thermodynamic and dynamic stabilities, and other competing phases. 

The last open question is whether the presently computed exfoliation energies can be used as descriptors of the isolated MXenes (0001) basal planes surface chemical activity. This is considered here using the CO_2_ adsorption energies, one of the most extended systems of study concerning carbon capture and storage, and DFT values previously obtained for a series of studies [17,19,38,39]. These studies were carried out with the same models and computational setups as the ones here employed, thus allowing a direct, consistent comparison, with the only caveat that the CO_2_ adsorption energies, $E_{ads}^{{CO}_{2}}$, were obtained using the PBE functional but also including the D3 dispersion contribution [40], i.e., PBE-D3. Notice that the inclusion of dispersion is reasonable for adsorbates, but it does not conflict with the description of bulk cells and surface slabs at the PBE level. This is because dispersion-driven interactions are negligible in bulk systems, although they might be important in the adsorption of the adsorbate. This is particularly the case when physisorption occurs, which is not the case for CO_2_ on MXenes, displaying consistently chemisorption situations. Notice that for each MXene compound, only the most stable adsorption configuration, i.e., the one with the most negative $E_{ads}^{{CO}_{2}}$ value, has been regarded for the analysis. 

**Figure 6:**
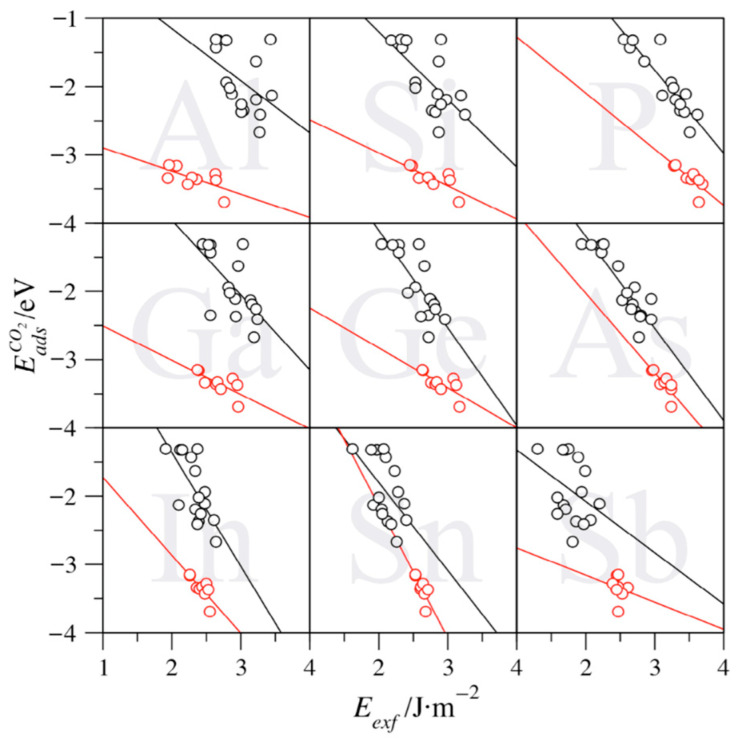
Trends of the CO_2_ adsorption energy ($E_{ads}^{{CO}_{2}}$) vs. the exfoliation energy (*E_exf_*) for C-based MXenes. Black circles denote groups V and VI MXenes altogether, while red ones denote group IV ones. The black and red lines are the linear adjustments of the studied values.

For each studied A element, Figure 6 shows the evolution of the $E_{ads}^{{CO}_{2}}$ in front of the obtained *E_exf_* for C-based MXene compounds cases. A clear, consistent trend is observed, insomuch the larger the exfoliation energy, the stronger the CO_2_ adsorption. Consequently, large *E_exf_* values imply a high chemical activity of the MXene surface. In other words, MXenes featuring larger exfoliation energies are more active, in the sense that they are less stable, and so are more prompt to adsorb atoms and/or molecules to compensate for their poor stability. However, a distinct behavior emerges for group IV MXenes when compared to those involving groups V and VI elements (see Figure 6). Group IV MXenes display a similar slope, yet the points are normally shifted toward larger $E_{ads}^{{CO}_{2}}$ while displaying smaller *E_exf_* values. This translates into group IV having smaller exfoliation energies, while stronger attaching the CO_2_, which could imply easier conditions of synthesizability while displaying an enhanced surface chemical activity. This outlying trend correlates with the fact that group IV MXenes are among the most commonly experimentally achieved [37]. This is particularly the case for Ti-based MXenes, and, at the same time, putting the accent on the possibilities of other group IV MXenes involving Zr or Hf.

Aside from the previous, one can highlight particular MAX phases where such a trend is most followed, particularly for P-based MAX phases (see Table 4), with a regression coefficient (*R*) above 0.9. For the other explored situations, the *R* values are smaller, and, even if the aforementioned trends are captured, one should not claim a linear relation and should rather note that exfoliation energies are a contributing factor to the surface chemical activity among other structural, energetic, or electronic features that may play a role as well. Indeed, the CO_2_ adsorption landscape on such MXenes is rich [17,19,38,39], and indeed the mixture of diverse sites in this analysis contributes to the dispersion of results, and, therefore, into a poorer linear regression. However, the same rich diversity does not allow a more detailed analysis of certain adsorption conformations, as the number of available cases is meager, with a concomitant meaningless statistical analysis. The slopes of the linear regression also quantify how dependent the CO_2_ adsorption energy is on the exfoliation energy; the ones for groups V and VI, ranging −0.75 eV/J·m^−2^ (Sb) to −1.67 eV/J·m^−2^ (In), being larger than those of group IV, being generally softer, from −0.34 (Ti) to −1.17 eV/J·m^−2^ (As), with the value of Sn of −1.94 eV/J·m^−2^ being a clear outlier. Besides, the intercepts serve to seize which part of the adsorption energy is affected by *E_exf_*, since one can argue that an MXene material with zero surface energy would have a likewise zero contribution to adsorb atoms or molecules upon. Within this argument, group V and VI MXenes reveal positive intercepts, which is in line with the aforementioned more negative slopes. Only for group IV MXenes, negative intercepts are found, from −0.58 eV (In) to −2.56 eV (Al), which implies that such MXenes display an inherent electronic structure that makes the system active toward CO_2_, modulated to some smaller extent by the surface energy instability compensation. Exceptions to this rule are found in the *p*^3^ elements P, As, and Sb. 

**Figure 7:**
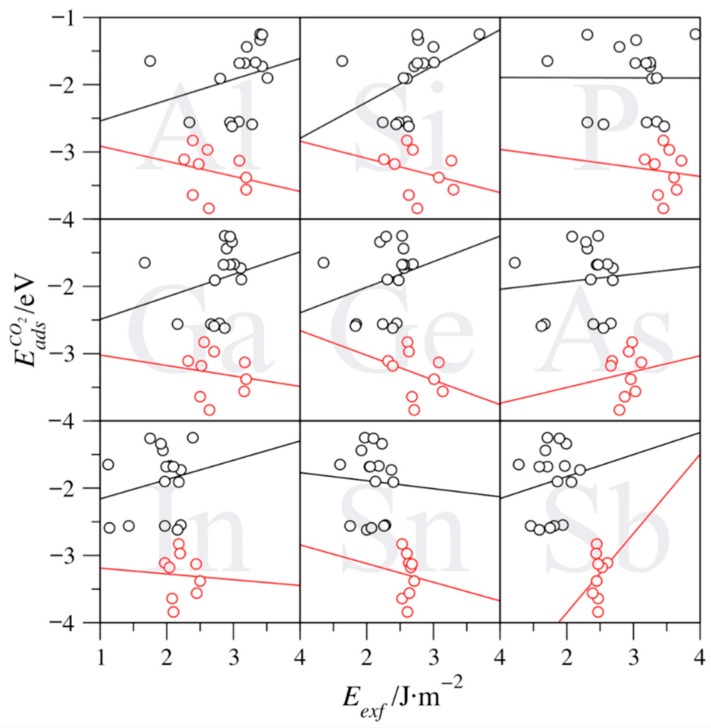
Trends of the CO_2_ adsorption energy ($E_{ads}^{{CO}_{2}}$) vs. the exfoliation energy (*E_exf_*) for N-based MXenes. Black spheres denote groups V and VI MXenes altogether, while red ones denote group IV ones. The black and red lines are the linear adjustments of the studied values.

The aforementioned trends are, however, lost whenever N-based MXenes are considered, as shown in Figure 7. The analysis reveals that the exfoliation energy effect is reversed for groups V and VI elements, except for Sn-based ones, which features a barely negative trend, with a slope of −0.12 eV/J·m^−2^ (see Table 4). Actually, the trends are minimal—up to 0.54 eV/J·m^−2^ (Si). At the same time, the intercepts are markedly negative—from −1.65 (Sn) to −3.34 eV (Si). For group IV MXenes, the slopes are generally negative, but also quite reduced—from −0.37 (Ge) to 0.24 eV/J·m^−2^ (As)—with Sb being a clear outlier. In any of the above cases, it seems clear that the effect of surface instability is sensibly reduced on N-based MXenes, which correlates with the overall lower *E_exf_* values for N-based MXenes compared to C-based ones, as above shown in Figure 3. Thus, on such MXenes, the details of the surface electronic structure are what govern the surface chemical activity rather than exfoliation energy. 

The above analysis underscores the different roles of the MAX parent phase exfoliation energies. Thus, the lowest *E_exf_* values are encountered when having N, W, or Sb, with *n* = 3, with W_4_SbN_3_ being the maximal exponent featuring, indeed a negative *E_exf_*, which implies instability of the MAX phase. On the other hand, the largest *E_exf_* values are found when the MAX phase involves C, Ti, and P, and for *n* = 1. Hence, Ti_2_PC becomes the target MAX phase, which, with an *E_exf_* of 3.64 J·m^−2^, is very close to the maximum computed estimate, belonging to Ti_2_PN, with an *E_exf_* of 3.72 J·m^−2^. Further than that, the exfoliation energy is found to be a factor defining the surface chemical activity, particularly for C-based MXenes, so that the larger the *E_exf_*, the larger the chemical activity. However, group IV MXenes display strong adsorptions and low exfoliation energies, at variance from what one would expect. Moreover, the N-based MXenes, which display lower *E_exf_* values, do not feature such a surface activity dependence. Altogether, such trends are valid rules-of-thumb so as to forecast future chemical activities on pristine MXenes or interpret results. This becomes particularly important given the recent experimental reports on cleaning protocols for terminated MXenes [18] and the novel synthetic routes capable of generating surface pristine MXenes [41].

## 4. Conclusions

Spurred by the successful employment of surfaces energies as physicochemical descriptors of a given surface chemical activity, we analyzed here whether exfoliation energies can be employed to predict the chemical activity of MXenes, which followed the fact that the bond strength between the *p*-block A element and the M early transition metal in a given MAX phase was found to be related to the easiness of MXene extraction. To further investigate this trend, the exfoliation energy was acquired for a total of 486 MAX phases exploring all the combination of M metals from groups IV–VI (Ti, Zr, Hf, V, Nb, Ta, Cr, Mo, W), X being C or N, and *p*-block A elements from groups XIII–XV (Al, Ga, In, Si, Ge, Sn, P, As, Sb), considering as well three M*_n+1_*X*_n_* for *n* = 1–3 thicknesses. 

The results reveal that N-based MXenes display lower exfoliation energies than the C-based counterparts. Moreover, as far as the M element is concerned, the *E_exf_* values decrease when going along the *d* series, with upside-downs along the groups. On the other hand, the *E_exf_* values decrease when going down a group for *p*-block elements, with oscillations along the *p* series. Concerning the number of atomic layers, it seems that the thicker the MXene, the lower the exfoliation energy, although with little variations. The analysis of the exfoliation energies for the cases experimentally realized revealed that most of MXenes are likely to be isolated, and, presumably, smoother conditions would be necessary, as far as the MAX and MXene phases are thermodynamically and dynamically stable. 

The exfoliation energies are found to have a significant effect on the chemical activity of the surface. Similar to what happens with surface energies, the exfoliation energy seizes the energetic toll to break the M–A bonds in the MAX phase. Thus, a large exfoliation energy implies a high-energy final MXene material, which will be more prone to create new bonds so as to reduce its intrinsic energy. Therefore, MXenes with larger exfoliation energies will exhibit more unstable MXene surfaces with a concomitant higher chemical activity and stronger bonding. This has been exemplified using CO_2_ adsorption energies on the explored MXenes, as this is related to carbon capture and storage technologies. The above-predicted trend is found to be particularly true for C-based MXenes, with a significant deviation of group IV MXenes, displaying stronger CO_2_ adsorption energies that one would expect from the rather small *E_exf_* values. On the contrary, N-based MXenes do not feature a dependence of the surface chemical activity, due to their natural higher stability, reflected on their smaller exfoliation energies. Altogether, the present study reveals that exfoliation energies can be a key property determining the MXenes stability and surface chemical activity with implications not only on MXene synthesizability aspects but also on the resulting MXene surface chemistry. 

## Figures and Tables

**Table 1:** Exfoliation energies (*E_exf_*) of all the studied M_2_AX MAX phases; values are given in J·m^−2^.

*E_exf_* M_2_AX/J·m^−2^											
X		M/A	Al	Si	P	Ga	Ge	As	In	Sn	Sb
**C**	***d*^2^**	**Ti**	2.76	3.16	3.64	2.96	3.17	3.24	2.55	2.68	2.47
		**Zr**	2.07	2.48	3.28	2.39	2.64	2.96	2.26	2.53	2.45
		**Hf**	2.36	2.74	3.53	2.64	2.82	3.08	2.40	2.60	2.44
	***d*^3^**	**V**	3.28	3.25	3.62	3.24	2.96	2.95	2.37	2.18	1.97
		**Nb**	2.87	2.85	3.45	2.92	2.74	2.95	2.48	2.37	2.20
		**Ta**	3.01	2.82	3.43	2.93	2.61	2.79	2.39	2.13	1.86
	***d*^4^**	**Cr**	3.45	3.19	3.11	3.14	2.76	2.53	2.10	1.92	1.68
		**Mo**	3.22	2.87	2.85	2.96	2.66	2.47	2.34	2.23	1.99
		**W**	3.43	2.90	3.08	3.03	2.58	2.26	2.37	2.07	1.75
**N**	***d*^2^**	**Ti**	3.09	3.27	3.72	3.17	3.08	3.12	2.44	2.31	2.16
		**Zr**	2.39	2.60	3.45	2.56	2.61	2.98	2.18	2.28	2.15
		**Hf**	2.61	2.69	3.54	2.71	2.63	2.93	2.20	2.15	1.91
	***d*^3^**	**V**	3.33	3.01	3.25	3.01	2.69	2.61	2.05	1.86	1.63
		**Nb**	3.43	2.71	3.25	3.11	2.56	2.69	2.21	2.12	1.96
		**Ta**	3.51	2.55	3.35	3.12	2.31	2.36	1.97	1.71	1.47
	***d*^4^**	**Cr**	3.20	3.00	2.79	2.90	2.55	2.31	1.94	1.81	1.54
		**Mo**	3.40	2.77	3.04	2.98	2.20	2.29	1.91	1.88	1.63
		**W**	3.28	2.44	2.55	2.71	1.83	1.62	1.14	1.01	0.77

**Table 2:** Exfoliation energies (*E_exf_*) of all the studied M_3_AX_2_ MAX phases; values are given in J·m^−2^.

*E_exf_* M_3_AX_2_/J·m^−2^											
X		M/A	Al	Si	P	Ga	Ge	As	In	Sn	Sb
**C**	***d*^2^**	**Ti**	2.63	3.01	3.56	2.88	3.08	3.17	2.50	2.64	2.39
		**Zr**	1.96	2.45	3.30	2.37	2.63	2.98	2.26	2.53	2.47
		**Hf**	2.29	2.71	3.59	2.66	2.84	3.14	2.44	2.61	2.47
	***d*^3^**	**V**	3.22	2.98	3.30	3.16	2.80	2.68	2.34	2.04	1.71
		**Nb**	3.04	2.77	3.37	3.04	2.72	2.80	2.61	2.40	2.07
		**Ta**	3.27	2.87	3.51	3.19	2.72	2.77	2.64	2.26	1.81
	***d*^4^**	**Cr**	3.06	2.77	2.84	2.84	2.47	2.28	1.97	1.75	1.46
		**Mo**	2.68	2.18	2.68	2.56	2.29	2.23	2.12	1.97	1.71
		**W**	2.64	2.32	2.55	2.45	2.04	1.94	1.91	1.62	1.30
**N**	***d*^2^**	**Ti**	3.19	3.30	3.65	3.16	3.14	3.03	2.45	2.37	2.00
		**Zr**	2.39	2.63	3.37	2.50	2.68	2.87	2.08	2.32	2.08
		**Hf**	2.63	2.76	3.45	2.64	2.71	2.79	2.10	2.23	1.84
	***d*^3^**	**V**	3.17	2.85	3.19	2.95	2.56	2.45	1.99	1.71	1.38
		**Nb**	2.80	2.60	3.28	2.72	2.48	2.69	2.18	2.05	1.79
		**Ta**	2.95	2.48	3.20	2.66	2.24	2.40	1.97	1.68	1.33
	***d*^4^**	**Cr**	2.34	2.24	2.31	2.16	1.84	1.67	1.43	1.25	0.95
		**Mo**	3.40	3.69	3.93	2.87	2.53	2.47	2.39	2.31	2.07
		**W**	3.22	2.42	2.52	2.63	1.75	1.44	0.71	0.53	0.26

**Table 3:** Exfoliation energies (*E_exf_*) of all the studied M_4_AX_3_ MAX phases; values are given in J·m^−2^.

*E_exf_* M_4_AX_3_/J·m^−2^											
X		M/A	Al	Si	P	Ga	Ge	As	In	Sn	Sb
**C**	***d*^2^**	**Ti**	2.64	3.03	3.64	2.95	3.12	3.24	2.53	2.71	2.45
		**Zr**	1.94	2.58	3.46	2.48	2.76	3.12	2.36	2.63	2.61
		**Hf**	2.23	2.79	3.69	2.71	2.90	3.24	2.48	2.66	2.53
	***d*^3^**	**V**	3.01	2.90	3.37	3.22	2.82	2.66	2.42	2.05	1.59
		**Nb**	2.79	2.53	3.24	2.82	2.53	2.71	2.48	2.28	1.94
		**Ta**	2.84	2.53	3.27	2.84	2.42	2.60	2.39	2.00	1.59
	***d*^4^**	**Cr**	2.84	2.52	2.55	2.66	2.28	2.10	1.91	1.60	1.28
		**Mo**	2.64	2.34	2.64	2.56	2.29	2.23	2.28	2.10	1.89
		**W**	2.79	2.40	2.68	2.53	2.20	2.08	2.15	1.89	1.67
**N**	***d*^2^**	**Ti**	3.19	3.08	3.61	3.19	3.01	2.96	2.50	2.32	1.88
		**Zr**	2.26	2.26	3.17	2.32	2.32	2.68	1.97	2.04	1.84
		**Hf**	2.48	2.42	3.32	2.52	2.39	2.66	2.04	1.94	1.63
	***d*^3^**	**V**	3.09	2.76	3.03	2.85	2.53	2.47	2.10	1.83	1.49
		**Nb**	3.08	2.60	3.35	2.79	2.45	2.66	2.21	1.94	1.75
		**Ta**	2.98	2.63	3.46	2.87	2.39	2.55	2.16	1.76	1.41
	***d*^4^**	**Cr**	1.75	1.63	1.71	1.67	1.35	1.22	1.12	0.83	0.53
		**Mo**	3.43	2.76	2.31	2.95	2.29	2.08	1.75	1.47	1.06
		**W**	3.20	2.34	2.39	2.56	1.67	1.30	1.20	0.50	−0.18

**Table 4:** Linear regression coefficients as displayed in Figure 6 and Figure 7, including slope (*a*) intersect, (*b*), and regression coefficient (*R*) according to the general equation $E_{ads}^{{CO}_{2}}$ = *a* · *E_exf_* + *b*.

X			Al	Si	P	Ga	Ge	As	In	Sn	Sb
**C**	**IV**	***a***	−0.34	−0.49	−0.82	−0.61	−0.70	−1.17	−1.14	−1.94	−0.40
		***b***	−2.56	−2.00	0.45	−1.72	−1.34	0.31	−0.58	1.73	−2.36
		***R***	0.65	0.76	0.76	0.78	0.79	0.78	0.77	0.74	0.15
	**V + VI**	***a***	−0.76	−0.99	−1.22	−1.10	−1.43	−1.35	−1.67	−1.29	−0.75
		***b***	0.38	0.78	1.89	1.23	1.75	1.50	1.97	0.77	0.57
		***R***	0.45	0.67	0.91	0.65	0.79	0.89	0.68	0.55	0.35
**N**	**IV**	***a***	−0.22	−0.25	−0.13	−0.16	−0.37	0.24	−0.09	−0.28	1.18
		***b***	−2.69	−2.59	−2.83	−2.87	−2.29	−3.97	−3.10	−2.57	−6.21
		***R***	0.25	0.28	0.00	0.16	0.32	0.11	0.05	0.05	0.22
	**V + VI**	***a***	0.31	0.54	0.00	0.33	0.38	0.11	0.29	−0.12	0.33
		***b***	−2.85	−3.34	−1.89	−2.83	−2.77	−2.15	−2.45	−1.65	−2.48
		***R***	0.28	0.45	0.07	0.24	0.26	0.09	0.21	0.05	0.15

## Data Availability

The data presented in this study are available on request from the corresponding author.
